# Synthesis and Evaluation of a Chitosan–Silica-Based Bone Substitute for Tissue Engineering

**DOI:** 10.3390/ijms232113379

**Published:** 2022-11-02

**Authors:** María I. Alvarez Echazú, Sandra J. Renou, Gisela S. Alvarez, Martín F. Desimone, Daniel G. Olmedo

**Affiliations:** 1 Facultad de Farmacia y Bioquímica, Universidad de Buenos Aires, Junín 956, Piso 3°, Buenos Aires C1113AAD, Argentina; 2 Cátedra de Química Analítica Instrumental, Junín 956, Piso 3°, Buenos Aires C1113AAD, Argentina; 3 Facultad de Odontología, Universidad de Buenos Aires, Cátedra de Anatomía Patológica, Marcelo T de Alvear 2142, Piso 2A, Buenos Aires C1122AAH, Argentina; 4 Facultad de Farmacia y Bioquímica, Instituto de Química y Metabolismo del Fármaco (IQUIMEFA), Universidad de Buenos Aires, Consejo Nacional de Investigaciones Científicas y Técnicas (CONICET), Junín 956, Piso 3°, Buenos Aires C1113AAD, Argentina; 5 Consejo Nacional de Investigaciones Científicas y Técnicas (CONICET), Godoy Cruz 2290, Piso 9, Buenos Aires C1425FQB, Argentina

**Keywords:** chitosan, silica, bone tissue engineering, biocomposite, murine experimental model

## Abstract

Bone defects have prompted the development of biomaterial-based bone substitutes for restoring the affected tissue completely. Although many biomaterials have been designed and evaluated, the combination of properties required in a biomaterial for bone tissue engineering still poses a challenge. In this study, a chitosan–silica-based biocomposite was synthetized, and its physicochemical characteristics and biocompatibility were characterized, with the aim of exploring the advantages and drawbacks of its use in bone tissue engineering. Dynamic light scattering measurements showed that the mean hydrodynamic size of solid silica particles (Sol-Si) was 482 ± 3 nm. Scanning electron microscopy of the biocomposite showed that Sol-Si were homogenously distributed within the chitosan (CS) matrix. The biocomposite swelled rapidly and was observed to have no cytotoxic effect on the [3T3] cell line within 24 h. Biocompatibility was also analyzed in vivo 14 days post-implant using a murine experimental model (Wistar rats). The biocomposite was implanted in the medullary compartment of both tibiae (*n* = 12). Histologically, no acute inflammatory infiltrate or multinucleated giant cells associated to the biocomposite were observed, indicating good biocompatibility. At the tissue–biocomposite interface, there was new formation of woven bone tissue in close contact with the biocomposite surface (osseointegration). The new bone formation may be attributed to the action of silica. Free silica particles originating from the biocomposite were observed at the tissue–biocomposite interface. According to our results, the biocomposite may act as a template for cellular interactions and extracellular matrix formation, providing a structural support for new bone tissue formation. The CS/Sol-Si biocomposite may act as a Si reservoir, promoting new bone formation. A scaffold with these properties is essential for cell differentiation and filling a bone defect.

## 1. Introduction

Bone is a specialized tissue composed of cells, fibers, and ground substances. In contrast to other connective tissues, the extracellular matrix in bone is mineralized, providing substantial strength and rigidity [1]. Although bone is a self-healing tissue, clinical intervention is required when a defect exceeds a certain size. Critically sized bone defects can be caused by trauma, tumor resection, and infection, among other factors [2], and are often treated using bone grafts or substitute biomaterials as therapeutic strategies [3,4]. 

Over the years, different biomaterials have been developed with the aim of achieving the properties and structure required for bone tissue engineering. Ceramics and biopolymers such as silica [5] and chitosan (CS) [6] have gained attention due to their biocompatibility. Although they are promising, these biomaterials alone can only mimic biological tissues to a limited extent, as occurs in the case of bone tissue. This is why there is currently research and development of new biocomposites that include both an organic and an inorganic component, with the aim of imitating natural bone tissue structure and function as faithfully as possible [7]. 

CS, which is plentiful in crustacean shells, is constituted of β−1,4-linked N-acetyl-D-glucosamine and D-glucosamine units. Biodegradable and non-toxic, CS is a well-known biomaterial for biomedical applications [8,9]. Moreover, its structural similarity with glycosaminoglycans has repeatedly been noted as an attractive feature for tissue engineering [10]. Indeed, glycosaminoglycans (GAGs) in the extracellular matrix have been reported to play an essential role in bone homeostasis, and potentially, regeneration [11]. Silica is plentiful in nature and has good biocompatibility. It is “generally recognized as safe” by the US Food and Drug Administration, and extensively used as a food additive and in manufacturing cosmetics [12]. Several in vitro and in vivo studies of silica-based materials have demonstrated that silica is biocompatible and has low toxicity [13], enabling its use as a biomaterial for bone tissue engineering. It has also been shown that silica nanoparticles can promote cell proliferation and differentiation, as well as bone mineralization [14]. 

Polymer–ceramic composites seem to be promising bone graft substitutes because natural bone is a composite consisting of collagen and hydroxyapatite [15]. Nevertheless, very few natural polymer–ceramic composites are commercially available, and those available are mainly based on type I collagen [16,17,18]. In this study, a CS–silica biocomposite was synthesized, and characterized physicochemically by scanning electron microscopy (SEM), dynamic light scattering (DLS) and Fourier transform infrared spectroscopy (FT-IR). Swelling and biodegradability properties were evaluated. A [3T3] mouse fibroblast cytotoxicity assay and an in vivo study were conducted to analyze its potential for bone tissue engineering. 

The aim of this study was to synthetize a CS–silica based biocomposite, characterize it physicochemically, and test it in an in vivo study.

## 2. Results

### 2.1. Ultrastructural Characterization of Sol-Si Particles and the CS/Sol-Si Biocomposite

**Figure 1:**
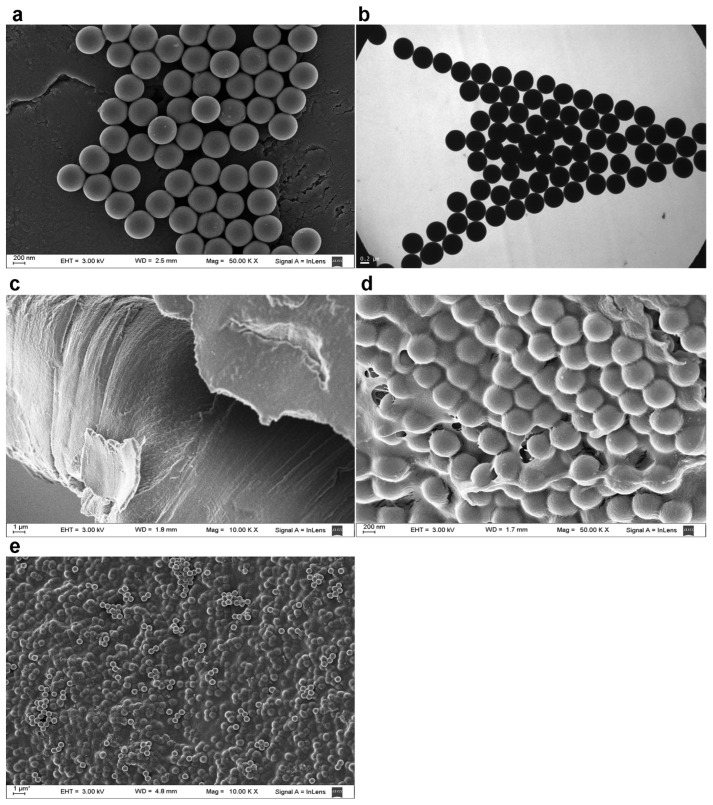
(**a**) Representative SEM image of Sol-Si particles; (**b**) representative TEM image of Sol-Si particles; (**c**) representative SEM image of CS; (**d**,**e**) representative SEM image of the (CS/Sol-Si) biocomposite.

In the Stöber method, monodisperse spherical silica nanoparticles are prepared by hydrolysis of tetraethyl orthosilicate (TEOS) followed by condensation in ethanol in the presence of ammonia [19]. This method tends to produce spherical colloidal silica of reasonably low polydispersity. This was corroborated for the synthesized particles by means of the DLS technique, and in SEM and TEM images, which showed that the Stöber silica particles were spherical, with no aggregation (Figure 1a,b). 

The mean hydrodynamic size and zeta potential ζ were determined by DLS. The zeta potential ζ of a colloidal particle is related to its surface charge. The mean hydrodynamic size of silica particles was 482 ± 3 nm, and zeta potential ζ was −43.6 ± 0.3 (Table 1). According to the results, silica spheres have negative zeta potential, reflecting the presence of deprotonated silanol groups on the surface [20]. 

The CS obtained by solvent casting had a compact structure with a smooth, spotless surface [21] (Figure 1c). Once the silica particles were incorporated, observation at high magnification showed that they were completely covered by the CS polymer matrix (Figure 1d). At lower magnification, the particles were homogenously distributed within the CS/Sol-Si biocomposite [22] (Figure 1e).

### 2.2. FT-IR Characterization

**Figure 2:**
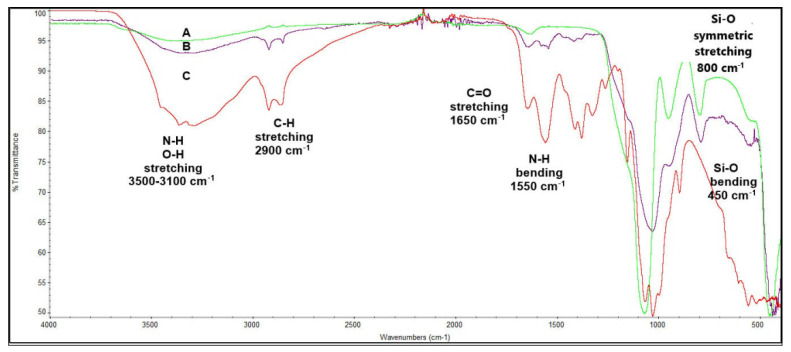
FT-IR IR Spectra. (A) Sol-Si particles, (B) CS/Sol-Si biocomposite, (C) CS.

The CS/Sol-Si biocomposite was characterized by FT-IR (Figure 2). The Sol-Si particles revealed characteristic IR bands in the region of 1050 cm^−1^ (Si-O-Si stretching), 800 cm^−1^ (Si-O symmetric stretching,) and 450 cm^−1^ (Si-O bending) [5]. CS showed a 1550 cm^−1^ (N-H) bending peak and a (C=O) stretching vibration peak at 1650 cm^−1^. There was a strong band in the region of 3500–3100 cm^−1^ corresponding to (N-H) and (O-H) stretching vibrations [23]. The peak at 2900 cm^−1^ corresponds to the (C-H) stretching vibration [24]. The CS/Sol-Si biocomposite revealed the characteristic IR bands of CS and Sol-Si described above, indicating that the composite is composed of both biomaterials.

### 2.3. Swelling Test

The water absorption behavior of a biocomposite is important for biomedical applications. Adequate water absorption is essential for nutrient diffusion throughout an implanted matrix [25]. Swelling also usually increases the surface area of the material, resulting in greater cellular attachment and infiltration [26]. 

**Figure 3:**
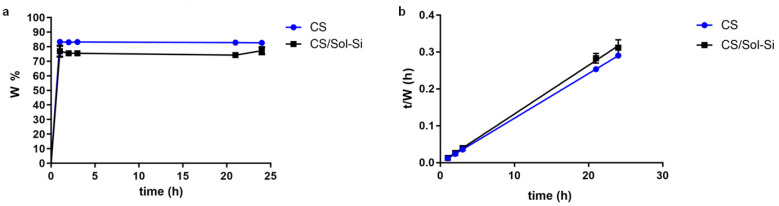
(**a**) Percentage of swelling of CS and CS/Sol-Si over a period of 24 h in PBS buffer. (**b**) CS and CS/Sol-Si experimental data for water content and time plotted according to a second-order kinetic model. Data were obtained from triplicate results and are shown as mean ± SD.

In the study, the CS and the CS/Sol-Si biocomposite rapidly absorbed a large quantity of water (Figure 3a). In both biomaterials, the swelling ratio reached equilibrium in 1 h. CS and CS/Sol-Si biocomposite water uptake followed second-order kinetics, as shown in Figure 3b. As specified in Table 2, CS water content at equilibrium was 82.6%. Once Sol-Si was added to the polymer matrix, water content at equilibrium decreased to 75.5%.

### 2.4. Contact Angle Measurements

**Figure 4:**
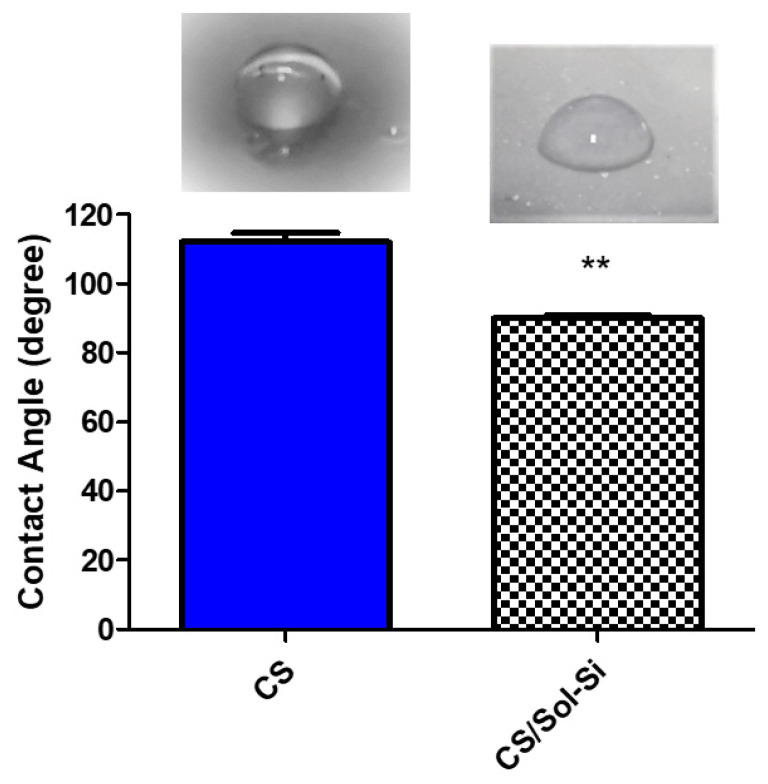
Contact angle of CS and CS/Si-Sol biocomposite. ** (*p* < 0.0001) according to Student’s *t*-test.

The chitosan film contact angle (110°) differed significantly from the composite contact angle (90°), indicating that silica particles impart a higher degree of hydrophilicity to the biomaterial. This is beneficial because the optimum values reported for promoting cell attachment and proliferation are 55° to 85° [27]. The contact angle is directly related to the wettability of the material and is therefore a predictive measure of the material’s cytocompatibility. The contact angle measured for the pure chitosan films was consistent with previously observed values, due to the hydrophobic nature of the chitosan backbone (Figure 4) [28]. 

### 2.5. Lysozyme Assay

Eight human chitinases have been identified to date, three of which possess enzymatic activity on CS. The biodegradation of CS forms non-toxic oligosaccharides of different lengths, which can be included in metabolic pathways or excreted [29].

CS degradation occurs primarily due to lysozyme and is regulated by the hydrolysis of the acetylated residues. The degradation rate depends on the level of crystallinity and acetylation. For instance, it has been shown that the biodegradation rate of highly deacetylated CS is slow [30]. These findings were expected due to the deacetylation percentage of CS employed. 

**Figure 5:**
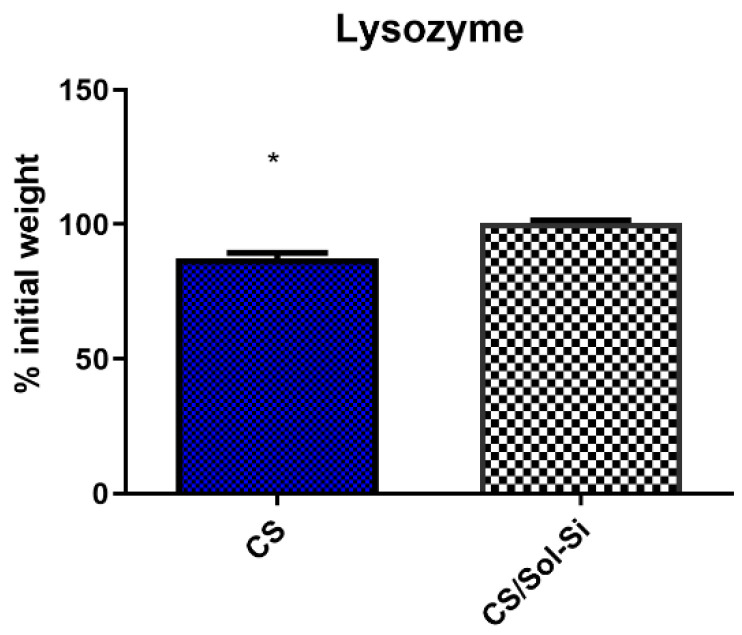
Biodegradation of CS and CS/Si-Sol biocomposite over 6 days with 1 mg/mL of lysozyme in PBS buffer, * (*p* < 0.05) according to Student’s *t*-test.

In this study, the in vitro degradation rate was higher for CS than for the CS/Sol-Si biocomposite, according to Student’s t-test (*p* < 0.05) (Figure 5). The biocomposite degradation rate was low over a period of 6 days. 

### 2.6. Cell Culture and Cytotoxicity Test

**Figure 6:**
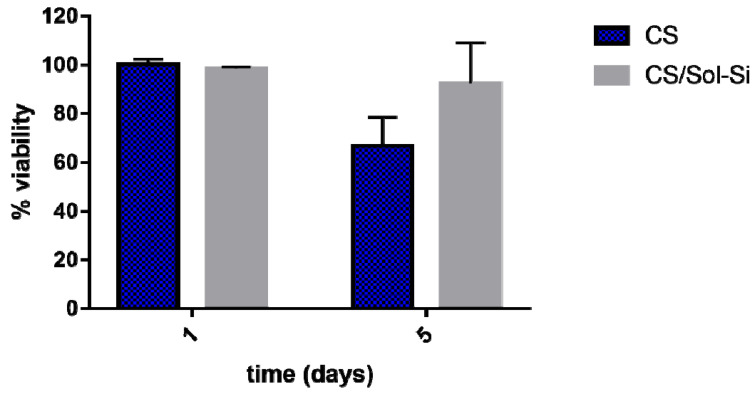
Percentage of viability of [3T3] mouse fibroblasts after incubation with CS and CS/Sol-Si at 1 and 5 days, by the MTT assay. Data are expressed as percentage relative to control. Data are shown as mean ± SD of triplicate experiments.

An in vitro cell cytotoxicity study was performed for 1- and 5-day incubation periods with a [3T3] mouse fibroblast cell line. A material’s cytotoxicity can be rated according to cell viability relative to controls, where a viability level of 90% relative to controls implies no cytotoxicity [31,32]. Considering this classification, CS and the CS/Sol-Si biocomposite were found to have no cytotoxic effect on the evaluated cell line. Both materials had a percentage of viability above 90%, implying that they are suitable for biomedical applications (Figure 6). They did not differ significantly according to Student’s *t*-test (*p* ≥ 0.05).

### 2.7. In Vivo Study

No change in animal body weight or behavior and no animal deaths were recorded during the experimental period.

#### Histological Analysis

**Figure 7:**
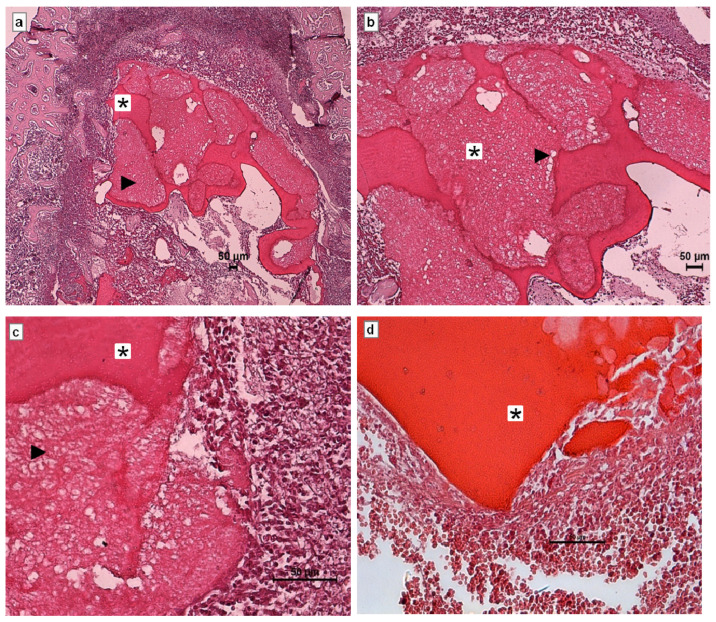
Histological evaluation of CS/Sol-Si biocomposite and pure chitosan (CS) at 1 day post-implantation in the hematopoietic bone marrow compartment. (**a**–**c**) Show the biocomposite composed of chitosan (*) and the silica particles (►). (**d**) shows only CS. All cases show the clot covering the entire surface of the biomaterial. (**a**) Orig. Mag. ×50; (**b**) Orig. Mag. ×100; (**c**,**d**) Orig. Mag. ×400. H–E stain. CS/Sol-Si (**a**–**c**). CS (**d**).

Histological evaluation of the CS/Sol-Si biocomposite and CS at 1 day post-implantation in rat tibia hematopoietic bone marrow compartment showed the presence of a clot covering the entire surface of the materials (Figure 7), indicating very good wettability of both biomaterials. There were also abundant polymorphonuclear neutrophils (PMNs) corresponding to acute inflammatory infiltrate, which is characteristic of the initial stages of a repair process.

**Figure 8:**
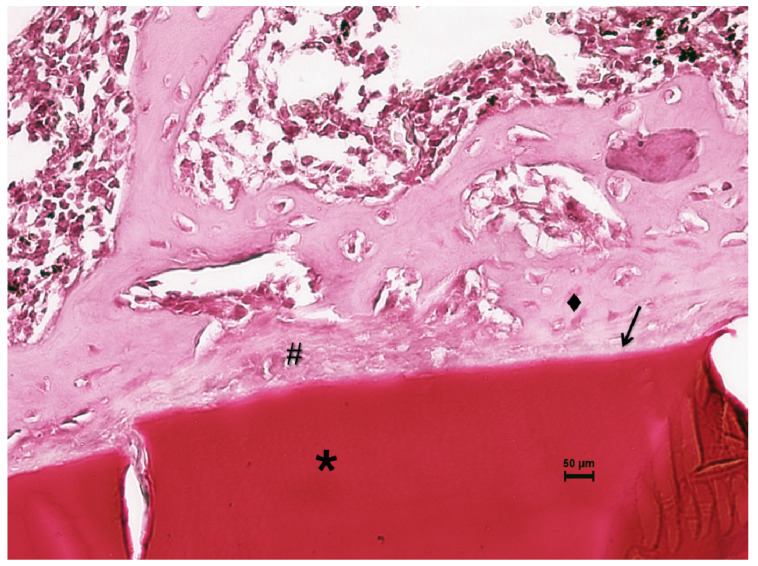
Histological evaluation of CS at 14 days post-implantation. At the tissue–biomaterial interface there is reparative granulation tissue (#) and formation of woven bone tissue (♦), sometimes in close contact with the surface of the biomaterial (osseointegration ↑). Orig. Mag. ×400; H-E stain. * Pure chitosan (CS).

**Figure 9:**
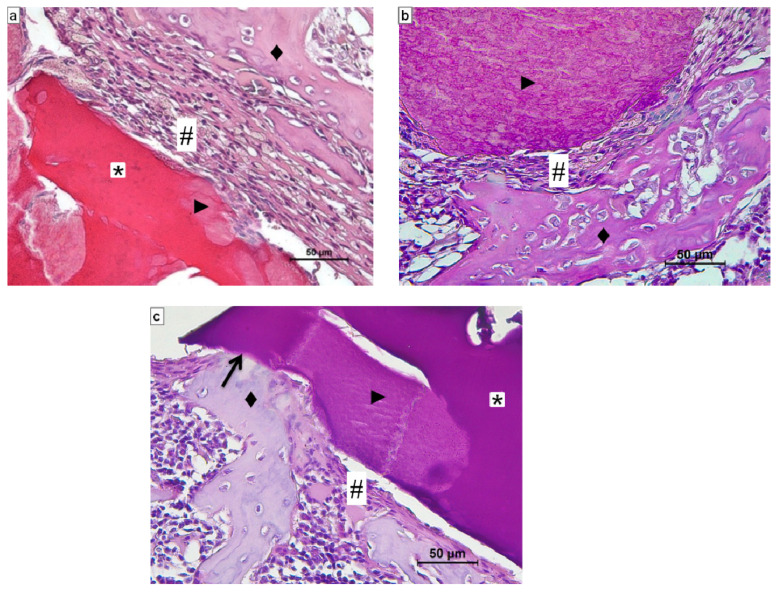
Histological evaluation of CS/Sol-Si biocomposite at 14 days post-implantation. (**a**,**b**) Show at the tissue–biomaterial interface, the replacement of the clot by reparative granulation tissue (#) and formation of woven bone tissue (♦). (**c**) Shows osseointegrated woven bone tissue (↑). (**a**–**c**) Orig. Mag. × 400; H-E stain. (*) chitosan; (►) silica particles.

Fourteen days after the implantation of CS/Sol-Si and CS, there were no acute inflammatory infiltrates or multinucleated giant cells, indicating good biocompatibility of both materials. At the tissue–biomaterial interface, the clot was replaced by reparative granulation tissue with abundant fibroblasts and a new formation of woven bone tissue (immature bone, confirmed by polarized light microscopy), sometimes in close contact with the surfaces of both biomaterials (osseointegration) (Figure 8 and Figure 9). It is worth highlighting that histologically, larger areas of osseointegration were observed with CS/Sol-Si than with CS.

**Figure 10:**
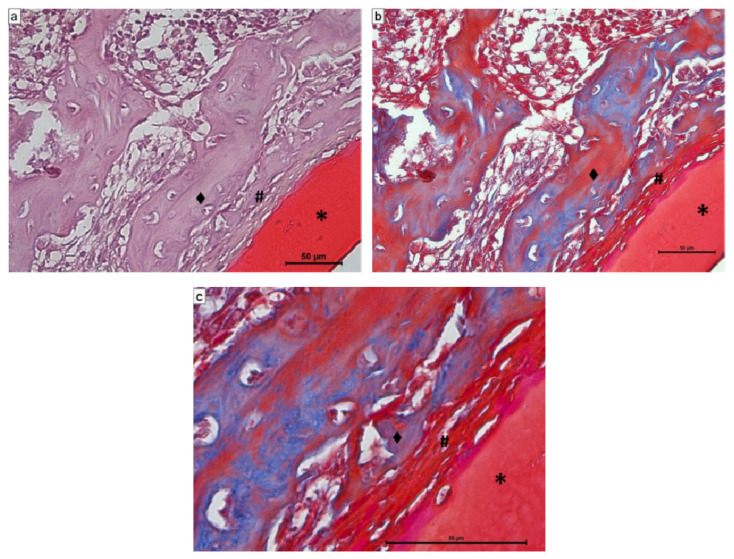
Histological evaluation of CS/Sol-Si biocomposite at 14 days post-implantation. At the tissue–biomaterial interface, there is reparative granulation tissue (#), which is progressively replaced by woven bone tissue (♦). Figures (**a**,**b**) correspond to the same image, (**a**) stained with H-E and (**b**) stained with Masson’s trichrome. (**c**) Higher magnification of part of image (**b**). Blue clearly shows the osteoid tissue that progressively replaces the reparative granulation tissue (red) next to the biomaterial (*). (**a**,**b**) Orig. Mag. × 400. (**c**) Orig. Mag. ×1000. (**a**) H-E stain. (**b**,**c**) Masson’s trichrome.

Masson’s trichrome stain clearly shows how the reparative granulation tissue (red) is progressively replaced by woven bone tissue (blue) (Figure 10b,c).

**Figure 11:**
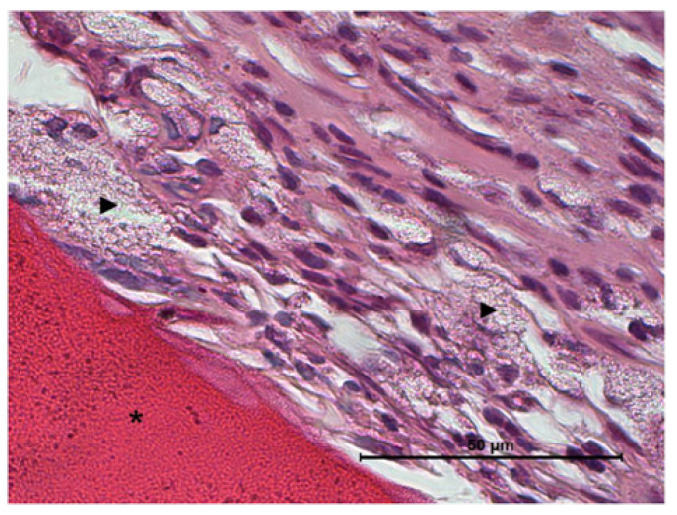
Histological evaluation of CS/Sol-Si biocomposite (*) at 14 days post-implantation. Silica particles (►) detached from within the biomaterial are observed at the tissue–biomaterial interface. No inflammatory infiltrate or multinucleated giant cells associated with the particle clusters are observed. Orig. Mag. ×1000; H–E stain.

**Figure 12:**
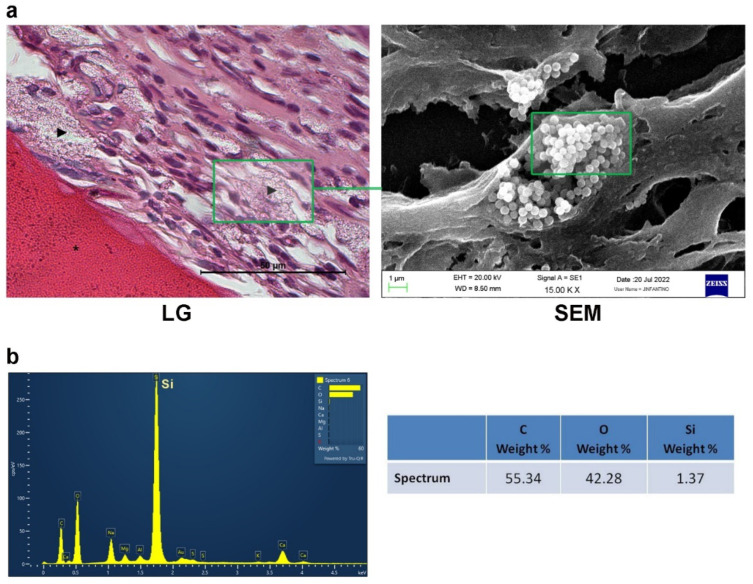
EDS analysis. (**a**) Area where analysis was performed at the level of the particles detached from the biomaterial. (**b**) EDS analysis of the particles showing the presence of Si. LG: light microscopy; SEM: scanning electron microscopy. Silica particles (►).

It is also worth highlighting that at the tissue–biocomposite (CS/Sol-Si) interface, there was the presence of particles, possibly originating from within the biomaterial, without the presence of inflammatory infiltrate or multinucleated giant cells associated with particle clusters (Figure 11). Microchemical analysis by EDS of the particles observed histologically confirmed the presence of silica in their composition (Figure 12).

## 3. Discussion

Bone defects are usually treated by grafting. However, grafting procedures are restricted by the limited amount of tissue, immune rejection, lack of biocompatibility, and the possibility of infection. A biomaterial-based scaffold is therefore a good strategy for treating bone defects, since it can act as a temporary frame and be inserted into the bone defect to support and induce bone tissue regeneration [26].

The use of CS or its composites as biomaterials for bone regeneration has been researched extensively [33,34], and there are constantly ongoing studies seeking to improve the applicability of such biomaterials to bone tissue repair. Different fillers, surface modifications, or synthesis methods are some of the approaches for enhancing the inherent properties of chitosan [35]. The current study combined Sol-Si particles and CS polymer to obtain a potential bone substitute. It has been demonstrated that silica particles have intrinsic biological activity, promoting osteoblast differentiation and inhibiting osteoclast differentiation [36]. Moreover, silica-based ceramics have been reported as an excellent resource for filling small bone defects. For bone substitution and repair, these ceramics are mainly used as fillers in non-load-bearing applications, and in coatings of metallic prostheses. It has also been suggested that they can be used for manufacturing ceramic–biopolymer scaffolds [37]. 

The aim of this study was to synthetize a CS–silica-based biocomposite, characterize it physicochemically, and test it in an in vivo study. A key point was the in vivo assessment of the biomaterial, to evaluate tissue response using an experimental model based on the osteogenic capacity of rat tibia bone marrow. This murine experimental model, developed by our research group [38], has been used to assess different systemic and local factors that influence tissue repair [39]. One particularity of this experimental model is that it provides a microenvironment free from microbial contamination and without mechanical forces, enabling objective assessment of the tissue response to the biomaterial, and ruling out confounding variables. It is important to highlight that for adequate bone repair, it is essential to immobilize the biomaterial at the surgical site. In our in vivo experimental model, this aspect is under control because throughout the duration of the experiment, the biomaterial is immobilized within the rat tibia bone marrow compartment, which serves as a three-dimensional scaffolding.

According to the SEM analysis, Sol-Si particles were distributed throughout the CS biopolymer matrix. DLS measurements showed that these Sol-Si particles had a mean hydrodynamic size of 482 ± 3 nm. This biocomposite may act as a template for cellular interactions and extracellular matrix formation, providing structural support for new tissue. A scaffold with these properties is vital to cell differentiation and filling a bone defect. The scaffold should be non-immunogenic, biocompatible, biodegradable, and non-toxic, since its structural properties not only influence cell survival, signaling, growth, and reorganization but also support the modulation of cell shape and gene expression [40]. 

The swelling studies showed that the composite absorbed a large quantity of water. Water content at equilibrium was ~75.5%. The swelling process promotes the loosening of coiled chitosan chains, ultimately leading to an increase in pore size and enhancing cell infiltration, nutrient distribution, and vascularization. Swelling can also increase the surface area, thereby promoting cell attachment and infiltration. The contact angle measurements also showed that biocomposites could favor cell colonization.

Depending on the technique used to obtain the CS and the composite, they may contain chitosan acetate. In this situation, as described by Mauricio-Sanchez et al. [41], chitosan amino groups are protonated (NH_3_^+^) that interact strongly with (-COO^-^) groups of acetic acid ions, leading to chitosan acetate. The remnant chitosan acetate may foster swelling, according to Chang et al. [42], who found that a neutralization step after solvent casting may decrease the swelling rate because of the increased chitosan hydrophobicity. This may explain the swelling behavior observed in our study. Moreover, Takara et al. [43] studied the effect of the neutralization of chitosan films against neutral aqueous media solubility. Their findings showed that the chitosan films obtained were water-soluble, whereas neutralized chitosan films were insoluble. According to this, the remaining chitosan acetate could be beneficial, since it may favor scaffold degradation, a desirable property for the synthesized biocomposite.

The degradation of the biocomposite was analyzed in vitro in a phosphate-buffered saline medium containing lysozyme and found to be low. However, it is worth highlighting that in an in vivo environment, the biocomposite would interact with many other factors which could affect its degradation. The degradation observed in vitro might therefore differ in vivo [26].

A cytotoxicity test was performed employing [3T3] mouse fibroblasts. As shown by the MTT assay, the CS–silica composite described above had no cytotoxic effect according to the classification proposed by Lönnroth et al. [31] and may therefore be considered as a potential biomaterial for biomedical applications.

Regarding histological evaluation, 1 day post-implantation, the clot was closely associated with the biocomposite, allowing it to be inferred that the material has adequate wettability. This biocomposite is highly wettable in a physiological environment because chitosan can absorb a large quantity of water and biological fluids due to its hydrophilic chains. Wettability has been extensively applied for designing dressing materials for wound healing or tissue engineering applications [44]. Wettability enables the rapid formation of hydrogel and colonization by formed elements of the blood in the scaffolding as from the time of implantation [45]. The histological findings of the current study soon after biocomposite implantation in rat tibia confirmed this phenomenon. Additionally, after one day, there were plentiful polymorphonuclear neutrophils (PMNn) corresponding to the acute inflammatory infiltrate, an expectable response during the first stages of any reparative process in relation to a biomaterial [46]. It is worth highlighting that in the histological assessment at 14 days post-implantation, no multinucleated giant cells (MNGCs) were found in relation to the biomaterial. Some authors call these cells biomaterial-associated multinucleated giant cells (BMGCs), and their implications in the inflammatory and reparative process are still unclear. BMGCs have frequently been associated to the foreign body giant cell type, formed from fusion of monocytes and macrophages in a process called “frustrated phagocytosis”, usually associated to the degradation of the biomaterial. BMGCs have long been considered pro-inflammatory cells per se [47,48]. However, in vitro and in vivo studies have shown that BMGCs present different phenotypic profiles depending on the physicochemical properties of the biomaterials to which they are associated and the expression of pro- and anti-inflammatory cytokines [48,49].

At 14 days post-implant, woven bone tissue and large areas of osseointegration were observed at the surface of the CS/Sol-Si biocomposite, but not at the surface of pure chitosan, where there was little osseointegration. The replacement over time of woven bone by laminar bone tissue will enable future histomorphometric evaluation of osseointegration. The histological results suggest that the silica particles contained in the chitosan may have promoted the formation of new bone tissue. In this regard, it has been demonstrated that Si is essential to connective tissue formation, osteoblast proliferation, and bone mineralization [50]. An in vitro study by Beck GR et al. [51] showed that silica nanoparticles (50 nm) stimulate mineralization and osteoblast differentiation, suppressing osteoclast differentiation, as well as improving in vivo bone mineral density. Our results show that the chitosan prepared using the method described was an optimum scaffold component of the biocomposite, promoting cell adhesion, cell proliferation, and new bone formation.

It is worth highlighting that finely particulate aggregates were observed at the biomaterial–tissue interface and in the bone marrow. According to the EDS study, these deposits corresponded to Si. Thus, as mentioned above, the CS/Sol-Si biocomposite may act as a Si reservoir, promoting bone repair after the chitosan has broken down and the particles have been released into the microenvironment. The literature contains no conclusive evidence on the cytotoxic, genotoxic, or carcinogenic effects of the breakdown of bone substitutes containing Si [52]. In the current study, Si particles did not form aggregates during SEM analysis of samples prior to implantation, though this phenomenon was observed when they were implanted in vivo in the hematopoietic bone marrow. When certain nanoparticulate materials are placed in a biological milieu, their aggregation state and surface area may change. Particle aggregation levels should be taken into account when the size and dose-dependent toxicity are considered [53]. It is well documented that particle aggregation in tissues triggers multinucleated giant cell (MNGC) recruitment [54]. However, neither MNGCs nor any other type of inflammatory infiltrates related to the aggregates was observed in the histological analysis of the samples at 14 days post-implantation. This may be due to factors such as particle size and distribution, state of aggregation, shape, crystalline structure, chemical composition, surface area and load, and porosity, which may condition the presence of inflammatory cells [55,56]. The silica particles formed aggregates in the tissues, appearing to act as “larger structures” with different surface roughness, such that the body may recognize the aggregates of microparticles. This could partly explain the lack of inflammatory response [57]. These results show that the particles have good biocompatibility.

CS can be used as a pure matrix biomaterial or combined with several types of structures, either embedded in the bulk material or deposited on the surface. As pure materials, biopolymers such as chitosan may have some limitations. For instance, many studies provide details of the biological functions of silica particles supporting bone cell adhesion, bone tissue formation, and biomineralization. Silica particles could therefore substitute the inorganic component of bone. However, they lack the organic component, which could be a polymeric template, and could aid the biological response, manipulation, and implantation of the biomaterial [58]. Inorganic and organic biomaterials have therefore been added to chitosan to serve either as a filler dispersed in the whole matrix, and/or as a coating at the material surface to enhance its properties [59]. The current study combined silica and chitosan to enhance their properties for bone tissue engineering purposes. Note that the mechanical properties of this biocomposite were not analyzed in this study because the biomaterial obtained could potentially be used in skeletal reconstructions such as small bone defects and/or regeneration of bone tissue in areas not exposed to excessive mechanical forces.

The CS/Sol-Si biocomposite designed and assessed using the in vivo experimental model presented herein showed good biocompatibility. However, further studies with longer experimental times are needed to assess tissue response in relation to its biological behavior and the replacement of the woven bone tissue by laminar bone tissue. Moreover, the presence of laminar bone tissue would enable histomorphometric evaluation of tissue response to the biomaterial. It is also important to conduct further studies to evaluate the ability of the silica particles observed in the bone marrow to promote bone tissue formation.

## 4. Materials and Methods

### 4.1. Materials

The materials were purchased from the following manufacturers (Table 3):

### 4.2. Synthesis of Solid Silica Particles (Sol-Si)

Solid silica particles (Sol-Si) were synthesized by the Stöber method [60]. In this synthesis, tetraethyl orthosilicate (TEOS) was used as a silicon precursor. The precursor was added to a solution of ammonium hydroxide in a water/ethanol mixture. All reagents were stirred with the aid of a magnetic stirrer at room temperature for 24 h, and silica particles of an approximate size of 500 nm were obtained. The particles were isolated by centrifugation and washed with distilled water until pH was neutral. The concentration of Sol-Si was determined by weighing the residual mass of an aliquot dried at 80 °C [61].

### 4.3. Synthesis of Chitosan–Silica Biocomposite (CS/Sol-Si)

Biocomposites were synthetized using a solvent casting technique. Briefly, a 20 mg/mL CS solution was prepared by dissolving chitosan powder in 1% (*v*/*v*) acetic acid. Solid silica particles were added to the solution while stirring to obtain a 50/50 CS/silica ratio. Then, 5 mL of CS/silica dispersion was poured into small Petri plates, used as templates. The solvent was evaporated at room temperature and the biocomposites were cut with a scalpel into 6.0 × 1.0 × 1.0 mm^3^ laminar implants. The same procedure was used without the Sol-Si particles to obtain CS implants. The implants were used to evaluate in vivo biological response and in vitro [3T3] mouse fibroblast cytotoxicity assay.

### 4.4. Ultrastructural Characterization of Sol-Si Particles and the CS/Sol-Si Biocomposite

Scanning electron microscopy (SEM) and transmission electron microscopy (TEM) were employed to analyze the shape and appearance of Sol-Si particles. Before analysis by the SEM technique (Zeiss SUPRA 40 microscope), CS/Sol-Si and CS were fixed in a glutaraldehyde solution (10% *v*/*v* in PBS) at 4 °C for 1 h, washed three times with PBS buffer, frozen at −80 °C, and afterward freeze-dried. The Sol-Si particles, CS/Sol-Si, and CS were all gold sputter-coated in an argon atmosphere before the analysis. The SEM images were registered at 50 k × and 10 k × magnification.

For TEM analysis (Zeiss 109 microscope), drops of Sol-Si particles in aqueous solution were placed on carbon-coated copper grids. After 1 min, the liquid was blotted with filter paper, and the particles were analyzed at room temperature. Before the measurements were performed, the aqueous solution was sonicated to ensure that the particles were suspended.

### 4.5. Fourier Transform Infrared Spectroscopy Characterization

The FT-IR spectra of the CS/Sol-Si, CS, and Sol-Si particles were determined at room temperature using an FT-IR-Raman Thermo Scientific (Waltham, MA, USA) Nicolet model 50 computer IS with a KBr beamsplitter with a resolution of 2 cm^−1^. All samples were measured using the attenuated total reflectance (ATR) technique. A background scan was recorded before measurement and subtracted from the sample spectra. The scanning range selected was 4000–500 cm^−1^, and the spectra collected were processed using Thermo Nicolet OMNIC software.

### 4.6. Swelling Study

The swelling behavior was evaluated as described in a previous publication [5]. Samples of the CS/Sol-Si and CS were freeze-dried, weighed (W_o_), and soaked in PBS buffer. At different times (t), the samples were removed from the buffer, wiped with bibulous paper, and weighed (W). The percentage of swelling was determined by the equation:

W% = [(W − W_o_)/W_o_] × 100

where W_o_ is the initial dry weight of the sample and W is the final weight of the sample.

Water content at equilibrium (W_∞_) and kinetic rate constants were determined by the equation:

t/W = 1/(KW_2 ∞_) + t/W_∞_

As described by Katime et al. [62], it is possible to calculate the water content at equilibrium (W∞) from a plot of t/W against time.

### 4.7. Contact Angle Measurements

Static water contact angle measurements were performed for pure water droplets using a drop volume of 20 µL. After the drop reached the surface, pictures were taken at a distance of 5 cm. The contact angle was calculated using contact angle plugin in ImageJ [63].

### 4.8. Lysozyme Biodegradability Test

CS and CS/Sol-Si biocomposites were freeze-dried and weighed (W_o_), after which they were immersed in PBS buffer (pH 7.4) with 1 mg/mL of lysozyme enzyme (100,000 U/mg) at 37 °C. After 6 days of incubation, the biomaterials were separated from the solution, washed with distilled water followed by freeze-drying, and weighed (W_6 days_). The percentage of weight was determined to evaluate biodegradation by the following equation:

W% = (W_6 days_/W_o_) × 100

### 4.9. Cell Culture and Cytotoxicity Test

First, [3T3] mouse fibroblast cells were grown in adherent culture flasks containing low-glucose Dulbecco’s modified Eagle medium (DMEM) with 1% penicillin-streptomycin and 10% heat-inactivated fetal bovine serum. They were incubated at 37 °C in a humidified 5% carbon dioxide chamber. Once confluence was attained, the harvest was conducted employing a trypsin-EDTA solution. Cells were stained with trypan blue and counted in a Neubauer chamber. Then, fibroblast cells (4.0 × 10^4^) were seeded in each well with 1 mL of complete low-glucose DMEM and incubated for 1 and 5 days. CS/Sol-Si and CS were placed on the fibroblast layer in the 24 wells, and the percentage of viability was measured after 24 h by an MTT assay. The medium was removed and replaced with 0.5 mL of 0.5 mg/mL MTT solution. The samples were incubated in a humidified 5% carbon dioxide chamber for 4 h. Afterwards, the MTT solution was removed, 1 mL of absolute ethanol was added, and they were incubated at room temperature for half an hour. Absorbance was measured at 570 nm in a UV–visible spectrophotometer (Cecil CE 3021, Cambridge, UK).

### 4.10. In Vivo Study

#### 4.10.1. Surgical Procedure

**Figure 13:**
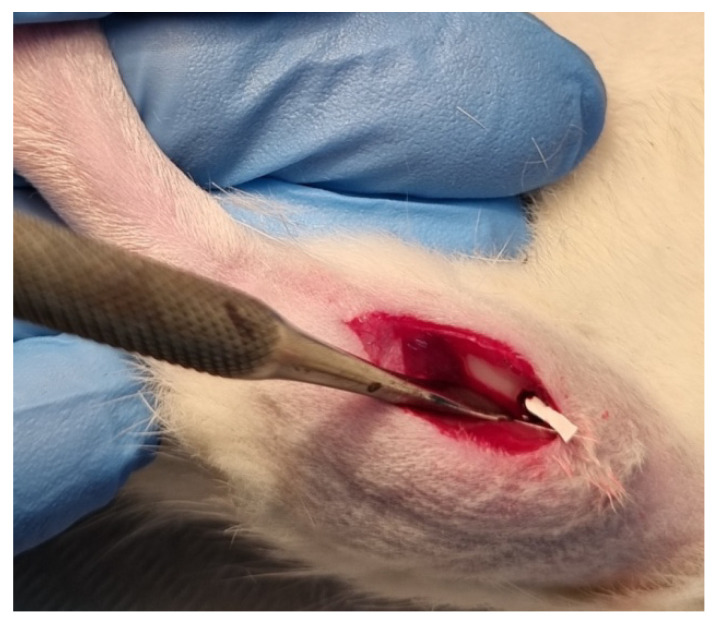
In vivo procedure: A laminar chitosan–silica biocomposite implant being placed through a hole in the tibial medullary compartment.

The biological response was evaluated experimentally in male Wistar rats (average body weight 120 g), fed ad libitum. One group (*n* = 8) had both tibias implanted with the biocomposite CS/Sol-Si, and another group (*n* = 4) with CS, was evaluated 14 days after implantation. A third group (*n* = 4) was implanted with both CS/Sol-Si and CS for evaluation at 24 h post-implantation. They were all operated under intraperitoneal anesthesia with a solution of 8 mg ketamine hydrochloride (Fort Dodge^®^, La Plata, Argentina) and 1.28 mg Xylazine (Bayer, Leverkusen, Germany) per 100 g of body weight. Both hind legs were shaved, and an incision was made along the tibial crest. The tissues were separated, exposing the tibial diaphyseal zone, and a hole 1.5 mm in diameter was made at the level of the bone cortex using manual rotation to prevent overheating and bone tissue necrosis. Laminar implants (6.0 × 1.0 × 1.0 mm^3^) were inserted in the hematopoietic bone marrow compartment of both tibias (*n* = 16), parallel to their long axis (Figure 13). The tissues were sutured. Antibiotic therapy was not administered because Wistar rats are resistant to infections. The biocomposite was decontaminated using 70° alcohol prior to surgery [38]. At the designated times after implantation, the rats were euthanized by anesthetic overdose. The tibiae were resected, radiographed, and fixed in 10% buffered formalin solution.

All procedures were conducted in accordance with the guidelines set forth by the National Institutes of Health (NIH Publication—Guide for the Care and Use of Laboratory Animals: Eighth Edition (2011) and the guidelines of the School of Dentistry, Buenos Aires University (Res (CD) 352/02 and Res (CD) 694/02) for care and use of laboratory animals. The protocol was approved by the School of Dentistry of Buenos Aires University’s Institutional Experimentation Committee (Resolution Number 009/2019-CICUAL-ODON-FOUBA).

#### 4.10.2. Histological Processing

The tibiae (*n* = 24) were demineralized in 10% ethylenediaminetetraacetic acid (EDTA, Anhedra, Argentina). After demineralization, the samples were embedded in paraffin, and cross-sections (10 µm) were cut at the level of the CS/Sol-Si or CS implant, stained with hematoxylin-eosin, and examined histologically under a light microscope (Leica, DM 2500, Wetzlar, Germany). Other histological sections were stained using Masson’s trichrome technique.

#### 4.10.3. Statistics

Data are presented as means ± SD of at least triplicate experiments, and Student’s t-test was used for the MTT assay. In the statistical evaluation, *p* < 0.05 was considered significant.

## 5. Conclusions

The chitosan obtained herein provided an optimum scaffold component of the biocomposite, promoting cell adhesion, proliferation, and new bone tissue formation. The CS/Sol-Si biocomposite may act as a Si reservoir, promoting new bone formation. The biomaterial obtained could potentially be used in skeletal reconstructions such as small bone defects and/or regeneration of bone tissue in areas not exposed to excessive mechanical forces.

## Figures and Tables

**Table 1:** DLS characterization of Sol-Si particles.

Hydrodynamic Size (nm) ± SD	PDI ± SD	Zeta Potential (mV) ± SD
482 ± 3	0.023 ± 0.020	−43.6 ± 0.3

**Table 2:** CS and CS/Sol-Si biocomposite water content at equilibrium (W_∞_) and coefficient of determination (r^2^) of water content and time plotted according to second-order kinetics.

	W_∞_	r^2^
CS	82.6	0.9999
CS/Sol-Si biocomposite	75.5	0.9993

**Table 3:** Materials.

~ Low-viscosity CS from crab shells, structural viscosity 66 mPas, deacetylation >75.0%, low molecular weight (<100 kDa). ~ Lysozyme from chicken egg white (100,000 U/mg) ~ MTT (thiazolyl blue tetrazolium bromide) reagent ~ TEOS (Tetraethyl orthosilicate) 98 wt%	Sigma-Aldrich (St. Louis, MO, USA)
~ Ammonium hydroxide solution 30%	Carlo Erba Reagents (Barcelona, España)
~ Dulbecco’s modified Eagle medium ~ Streptomycin ~ Penicillin ~ Fetal bovine serum	Gibco (New York, NY, USA)

All the other reagents were of analytical grade.

## Data Availability

Not applicable.
